# Chronic Activation of Heme Free Guanylate Cyclase Leads to Renal Protection in Dahl Salt-Sensitive Rats

**DOI:** 10.1371/journal.pone.0145048

**Published:** 2015-12-30

**Authors:** Linda S. Hoffmann, Axel Kretschmer, Bettina Lawrenz, Berthold Hocher, Johannes-Peter Stasch

**Affiliations:** 1 Pharma Research Centre, Bayer HealthCare, Wuppertal, Germany; 2 Instute of Nutritional Science, University of Potsdam, Potsdam, Germany, and IFLb Laboratoriumsmedizin Berlin GmbH, Berlin, Germany; 3 School of Pharmacy, Martin-Luther-University, Halle an der Saale, Germany; University Medical Center Utrecht, NETHERLANDS

## Abstract

The nitric oxide (NO)/soluble guanylate cyclase (sGC)/cyclic guanosine monophasphate (cGMP)-signalling pathway is impaired under oxidative stress conditions due to oxidation and subsequent loss of the prosthetic sGC heme group as observed in particular in chronic renal failure. Thus, the pool of heme free sGC is increased under pathological conditions. sGC activators such as cinaciguat selectively activate the heme free form of sGC and target the disease associated enzyme. In this study, a therapeutic effect of long-term activation of heme free sGC by the sGC activator cinaciguat was investigated in an experimental model of salt-sensitive hypertension, a condition that is associated with increased oxidative stress, heme loss from sGC and development of chronic renal failure. For that purpose Dahl/ss rats, which develop severe hypertension upon high salt intake, were fed a high salt diet (8% NaCl) containing either placebo or cinaciguat for 21 weeks. Cinaciguat markedly improved survival and ameliorated the salt-induced increase in blood pressure upon treatment with cinaciguat compared to placebo. Renal function was significantly improved in the cinaciguat group compared to the placebo group as indicated by a significantly improved glomerular filtration rate and reduced urinary protein excretion. This was due to anti-fibrotic and anti-inflammatory effects of the cinaciguat treatment. Taken together, this is the first study showing that long-term activation of heme free sGC leads to renal protection in an experimental model of hypertension and chronic kidney disease. These results underline the promising potential of cinaciguat to treat renal diseases by targeting the disease associated heme free form of sGC.

## Introduction

The second messenger cyclic guanosine monophosphate (cGMP) is generated by the heterodimeric α/β-heme protein soluble guanylate cyclase (sGC) upon activation by its endogenous ligand nitric oxide (NO) [1]. NO binds to the reduced prosthetic heme group bound to the β subunit [2]. cGMP is a key mediator of the cardiovascular system and its effects lead to vasodilation, inhibition of smooth muscle proliferation, blockade of leukocyte infiltration and inhibition of platelet aggregation [3–5]. Impairment of the cytoprotective NO/sGC/cGMP-signalling pathway is associated with the development of serious cardiovascular diseases such as hypertension or heart failure [6]. The systemic consequences of cardiovascular diseases represent the leading causes of death in the developed as well as developing countries [7]. Oxidative stress is associated with several cardiovascular diseases and is characterized by increased formation of reactive oxygen species (ROS) [8, 9]. ROS interfere with the NO/sGC/cGMP-signalling pathway via i) increased ROS production by NADPH-oxidases and uncoupled NO-synthases [10, 11], ii) scavenging of NO via reaction of NO and O_2_^-^ to peroxynitrite [12] and iii) oxidation and subsequent loss of the prosthetic heme group, the NO-binding site, from sGC [13]. The heme free form of sGC is unresponsive to NO and prone to upiquitin-mediated degradation [14–16].

The heme and NO-independent sGC activator cinaciguat (BAY 58-2667) stabilizes heme free sGC and thereby prevents its oxidation-induced degradation [15, 16]. Cinaciguat binds to the heme anchoring residues Y135 and R139 which are uncovered upon heme loss [17]. The compound is a heme mimetic and replaces the native prosthetic group resulting in similar conformational changes as observed upon binding of NO [17]. Cinaciguat-induced activation of enzyme preparations is increased up to 200-fold upon oxidation of the heme group with the sGC inhibitor ODQ compared to control conditions [18]. Interestingly, a similar activation was observed with the heme free enzyme where the orphaned heme binding pocket is occupied by cinaciguat [18–21]. Importantly, the concept of selective activation of heme free sGC by cinaciguat also applies to the *in vivo* situation: Cinaciguat more potently relaxes vessels of aged spontaneously hypertensive rats, hyperlipidemic rabbits and mice as well as arteries of type 2 diabetic patients than the respective control vessels [14]. In fundus and colon strips from mice expressing heme free variants of sGC, cinaciguat efficiently induced relaxation [22]. Furthermore, the blood pressure lowering effect of cinaciguat was stronger and prolonged in spontaneously hypertensive rats as compared to normotensive controls [14]. Cinaciguat improved cardiac output and renal blood flow in a model of congestive heart failure [23, 24]. Treatment of experimental chronic renal failure with cinaciguat resulted in beneficial renal and cardiovascular effects [25]. With the use of cinaciguat it became evident that virtually all enzyme preparations, cells and tissues bear a pool of heme free sGC and that this pool is increased under pathological conditions such as hypertension [14, 17, 20, 26–29]. The unique property of sGC activators such as cinaciguat to selectively activate heme free sGC allows to target the disease associated form of sGC and renders cinaciguat a promising pharmacological agent to treat oxidative stress associated diseases like hypertension.

Here, we investigated whether chronic activation of heme free sGC with cinaciguat results in a therapeutic effect in a model for salt-sensitive hypertension. This condition is associated with increased oxidative stress and hence increased levels of heme free sGC. Dahl/ss rats were fed a high salt diet (containing 8% NaCl) with or without cinaciguat for 21 weeks. This rat strain develops severe hypertension upon high salt intake with associated lesions in kidney and heart [30]. Furthermore, these pathological aberrations are accompanied by increased oxidative stress and, hence, by an increased pool of heme free sGC. Therefore Dahl/ss rats provide a good model to investigate the effects of chronic activation of heme free sGC by cinaciguat. Cinaciguat treatment resulted in significantly improved survival, significantly blunted increase in blood pressure and heart rate, improved renal function and reduced cardiac and renal inflammation and fibrosis. Our results show that cinaciguat has a high therapeutic potential for renal protection in hypertension.

## Material and Methods

### Chemicals

The sGC activator cinaciguat BAY 58-2667 ((4-[((4-carboxybutyl){2-4-phenethylbenzyl)oxy]phenethyl} amino)methyl-[benzoic]acid) was synthesized as described previously [18]. All other chemicals were of analytical grade and obtained from Sigma (Steinheim, Germany).

### Animal studies

#### Ethics statement

Animal studies were carried out in accordance with local ethical regulations for the use of laboratory animals. The studies were conducted according to the German animal welfare law and permitted by the Landesamt für Natur, Umwelt und Verbraucherschutz (LANUV) Nordrhein-Westfalen, Germany.

#### Study design

Male Dahl/ss rats (SS/JrHsdMcwiCrl, Charles River, Wilmington MA, USA) at the age of 7 weeks weighing 335–345 g were randomly allocated into two groups: placebo group (n = 20) receiving a high salt diet containing 8% NaCl, cinaciguat group (n = 17) receiving 1000 ppm cinaciguat in the solid feed of the high salt diet. All animals were housed under controlled standard conditions (12 h light and 12 h dark, at 24 ± 1°C) and received food and water *ad libitum*. The health status of the animals was assessed daily by well trained and experienced personnel. In case an animal was unwell and showed severe signs of suffering or distress such as weight loss, signs for pain or unresponsiveness the animal was sacrificed. Animals were sacrificed by CO_2_ asphyxiation if severe clinical signs of distress and disease such as 20% weight loss, lethargy and paralysis were observed. In the majority of the animals which died before study end such signs were not observed and the animals were found dead in the cages in the morning. To the best of our knowledge, the animals died due to the manifestation of the cardio-renal disease without apparent changes in behavior or appearance of the animals. Death was not the endpoint of our study. The endpoint analysis was performed on cardiac and renal function and pro-fibrotic and pro-inflammatory biomarkers (see below). The study was terminated in week 21 after half of the animals in the placebo group had died due to manifestation of the cardio-renal disease Dahl/ss rat develop upon feeding a high salt diet. Histopathological examination of descedent animals was performed as far as possible in 9 animals of the placebo groups and 2 animals of the cinaciguat group.

During the study the animals were weighed every third week. In week 19, 6 h urine samples were collected. Systolic blood pressure and heart rate were assessed via the tail-cuff method once before the study start and in week 6, 9, 12, 15 and 21 [31]. At the end of the study, animals were anesthetized with isoflurane and blood samples were taken. Afterwards animals were sacrificed by cervical dislocation free of pain. Heart, left ventricle and both kidneys were removed and weighed. Left ventricle and one pole of the left kidney (containing cortex and medulla) were snap frozen in liquid nitrogen and stored at -80°C until RNA was extracted. Lungs were perfused with formalin and processed to histopathological examination. Hearts were retained in formalin, right kidneys were fixed in Davidson solution (40% ethanol (96%), 20% formalin, 10% acetic acid, 30% H_2_O) and subjected to histopathological studies. Blood samples were centrifuged (4°C, 4000 rpm) and plasma was aliquoted and stored at -20°C until further analysis.

### Analysis of plasma parameters

Creatinin, urea, uric acid, creatine kinase (CK), lactate dehydrogenase (LDH), glutamate dehydrogenase (GLDH), aspartate amino transferase (AST), alanine amino transferase (ALT), alkaline phosphatase (AP), protein and albumin (ALB) were analyzed using a Multichannel Autoanalyzer (Synchron CX7, Beckmann, Hamburg, Germany) as previously described [32]. Atrial natriuretic peptide (ANP) and cGMP were extracted and analyzed using commercially available radioimmuno assay kits as described [33]. BNP levels in the plasma samples were below the detection limit of 0.05 ng/ml of the RIA Kit used in the study (BNP-45 (Rat)—RIA Kit, Phoenix Europe GmbH, Karlsruhe, Germany). Plasma concentrations of osteopontin (OPN) were analyzed using the Quantikine^®^ Mouse&Rat Osteopontin Immunoassay (R&D Systems, Minneapolis, MN, USA) according to the manufacturer’s instructions. Plasma concentrations of cinaciguat were measured using HPLC (2300 HPLC System, Cohesive Technologies, Thermo Fischer Scientific, Waltham, MA, USA) as described elsewhere [34].

### Analysis of urine parameters

Concentrations of creatinin, urea and protein were analyzed using the Multichannel Autoanalyzer [32]. Sodium and potassium were measured using an electrolyte analyzer (Instrumentation Laboratory, Bedford, MA, USA; [30]). Urinary concentrations of osteopontin and cGMP were analyzed as described for plasma samples.

### Determination of relative gene expression of pro-inflammatory and pro-fibrotic biomarkers

To determine the relative gene expression of the pro-fibrotic biomarkers collagen1α1 (*Col1α1*), fibronectin-1 (*Fn1*), osteopontin (*Spp1*), tenascin-C (*Tnc*) and kidney injury molecule-1 (*Kim1*) and the pro-inflammatory biomarker monocyte chemoattractant protein-1 (*Mcp1*) poles of the kidney and the left ventricle were minced in liquid nitrogen. Control organs from Dahl/ss rats (n = 10) of the same age receiving a diet with normal salt content were processed in parallel to determine relative gene expression in normotensive animals. mRNA was isolated using TRIzol^®^ (Invitrogen, Karlsruhe, Germany) and reversely transcribed using ImProm II Reverse Transcription System (Promega, Mannheim, Germany) according to the manufacturer’s instructions. Relative gene expression was determined using the TapMan^®^ method and specific primers and Taqman probes (Table 1). Samples were prepared in qPCR Master Mix Plus (Eurogentech, Seraing, Belgium) and quantitative Real-time-PCRs were performed with the 7900 HT Fast Real-Time PCR System (Applied Biosystems, Foster City, CA, USA). Relative gene expression was calculated using the ΔΔ-Ct-Method and ribosomal protein L32 (RpL32) as reference gene [35].

**Table 1:** Sequences of primers and TaqMan probes used for determination of relative gene expression of biomarkers.

Biomarker	Forward primer	Reverse primer	TaqMan probe
Col1α1	5’-gaagcatgtctggtttggagaga-3’	5’-atcggaaccttcgcttccat-3’	5’-tgaccgatggattccagttcga-3’
Mcp1	5’-tgcagttaatgccccactca-3’	5’-tctccagccgactcattgg-3’	5’-ctgctgctactcattcactggcaagatga-3’
Fn1	5’-ctgcacatgtctcgggaatg-3’	5’-cccgtcgtcataacacgttg-3’	5’-aaagggagaattcaaatgcgatcccc-3’
Spp1	5’-agccatgaccacatggacga-3’	5’-gattcgtcagattcatccgagt-3’	5’-agaccatgcagagagcgaggattctgtg-3’
Tnc	5’-tctgtggatggtacagtcaag-3’	5’-atgggctcaggtctgccagg-3’	5’-aagtcattgtggggcctgacaccacctcc-3’
Kim1	5’-ggaatggcactgtgacatcct-3’	5’-ctgcggcttcctcaaagg-3’	5’-aggaggcctggaataatcacactgtaaga-3’
RpL32	5’-gaagagcagcacagctggc-3’	5’-tcattctcttcgctgcgtagc-3’	5’-tcagagtcaccaatcccaacgcca-3’

### Echocardiographic studies

High resolution echocardiography was performed in week 20. Transthoraric echocardiography was performed after anesthesia with isoflurane (2–2,5%) using Sequia 512 Equipment (Acuson, Mountain View, CA, USA) equipped with a 15 MHz linear array transducer placed gently on the shaved right hemithorax. Parasternal left ventricular end-systolic and end-diastolic diameters and wall thickness were measured in the short axis view at the level of the papillary muscles using two dimensional guided M-mode imaging [36]. The measurements were conducted in accordance with the recommendations of the American Society of Echocardiography. Fractional shortening (FS) was calculated using the equation: FS (%) = ((LVDd-LVDs)/LVDd)*100 (LVDd: left ventricular dimension at end-diastole; LVDs: left ventricular dimension at end-systole). All measurements were performed offline in DICOM—format by using SonoWin 4.1.6 (Meso International GmbH, Mittweida, Germany).

### Histopathological studies

Tissue samples from fixed hearts, lungs and right kidneys were embedded in paraffin (Paraplast^®^, Carl Roth GmbH & Co. Kg, Karlsruhe, Germany). Organs from animals which died before study end (placebo group n = 9; cinaciguat group n = 2) were removed as well and prepared for histological examination. Embedded tissue samples were cut into 3 μm sections and subjected to hematoxylin-eosin (HE) staining. Sections of kidneys were additionally stained with Periodic Acid Schiff (PAS). The microscopical examination comprised a semi-quantitative analysis using a score 1–5 (minimal-slight-moderate-marked-massive). The histopathological findings were entered online into the PathData^®^ computer program version 6.2c2 (PathData is a registered trademark of Pathology Data Systems, Inc., Mt. Arlington, NJ, USA). In order to discriminate among different group sizes, the incidences are displayed in percent.

### Statistics

Data are means ± SEM. Statistic comparisons were performed using the unpaired Student’s *t*-test and the Chi square (Γ^2^) test. The level of significance was set at p<0.05. A one-sided exact Fisher Test was used for histopathology data.

The ARRIVE Guidelines Checklist is shown in S1 ARRIVE Checklist.

## Results

### *In vivo* parameters

**Fig 1:**
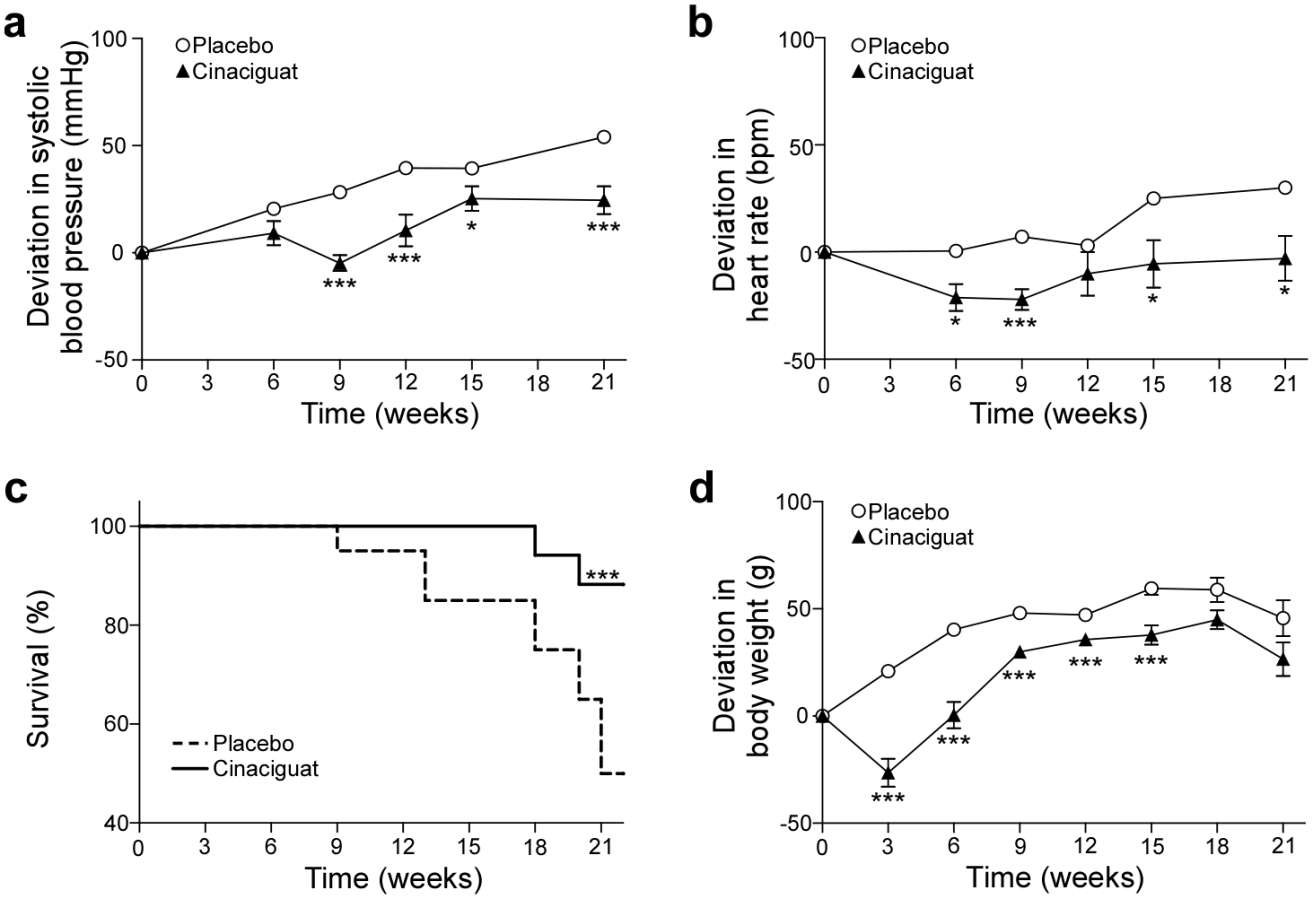
*In vivo*-parameters. Relative effects of oral treatment with cinaciguat on systolic blood pressure (a), heart rate (b), survival (c) and body weight (d) of Dahl/ss rats on high salt diet (8% NaCl). Data are shown as means ± SEM. Initial values of systolic blood pressure were 176.7 ± 3.6 mmHg and 182.8 ± 3.5 and heart rate were 409.4 ± 3.6 bpm and 407.2 ± 4.9 bpm in the placebo and the cinaciguat group, respectively. At beginning of the study animals weighed 335.5 ± 5.1 g and 339.9 ± 3.9 g in the placebo and the cinaciguat group, respectively. *p < 0.05; ***p < 0.005: Student’s *t*-test (cinaciguat vs. placebo).

Blood pressure increased 54.0 ± 8.5 mmHg in the placebo group whereas a significantly lower blood pressure increase of only 24.5 ± 6.5 mmHg was observed in the cinaciguat-treated rats (Fig 1a). Concomitantly, heart rate increased 30.0 ± 10.7 bpm in the placebo group and was significantly lower in the cinaciguat group (Fig 1b).

Importantly, cinaciguat highly significantly improved survival (Χ^2^ = 8) compared to placebo (Fig 1c). In the placebo group 10 out 20 animals died within 21 weeks after commencement of treatment. Until the end of the study only two animals died in the cinaciguat group in week 18 and 20, respectively, corresponding to a survival rate of 82%.

Animals of both groups had similar body weights at the beginning of the study (Fig 1d). During the course of the study animals of the placebo group gained weight continuously. At the beginning of the study food intake was reduced in the cinaciguat-treated group presumably due to the taste of the food. After animals adapted to the taste, food intake and weight gain increased.

### Laboratory results

**Fig 2:**
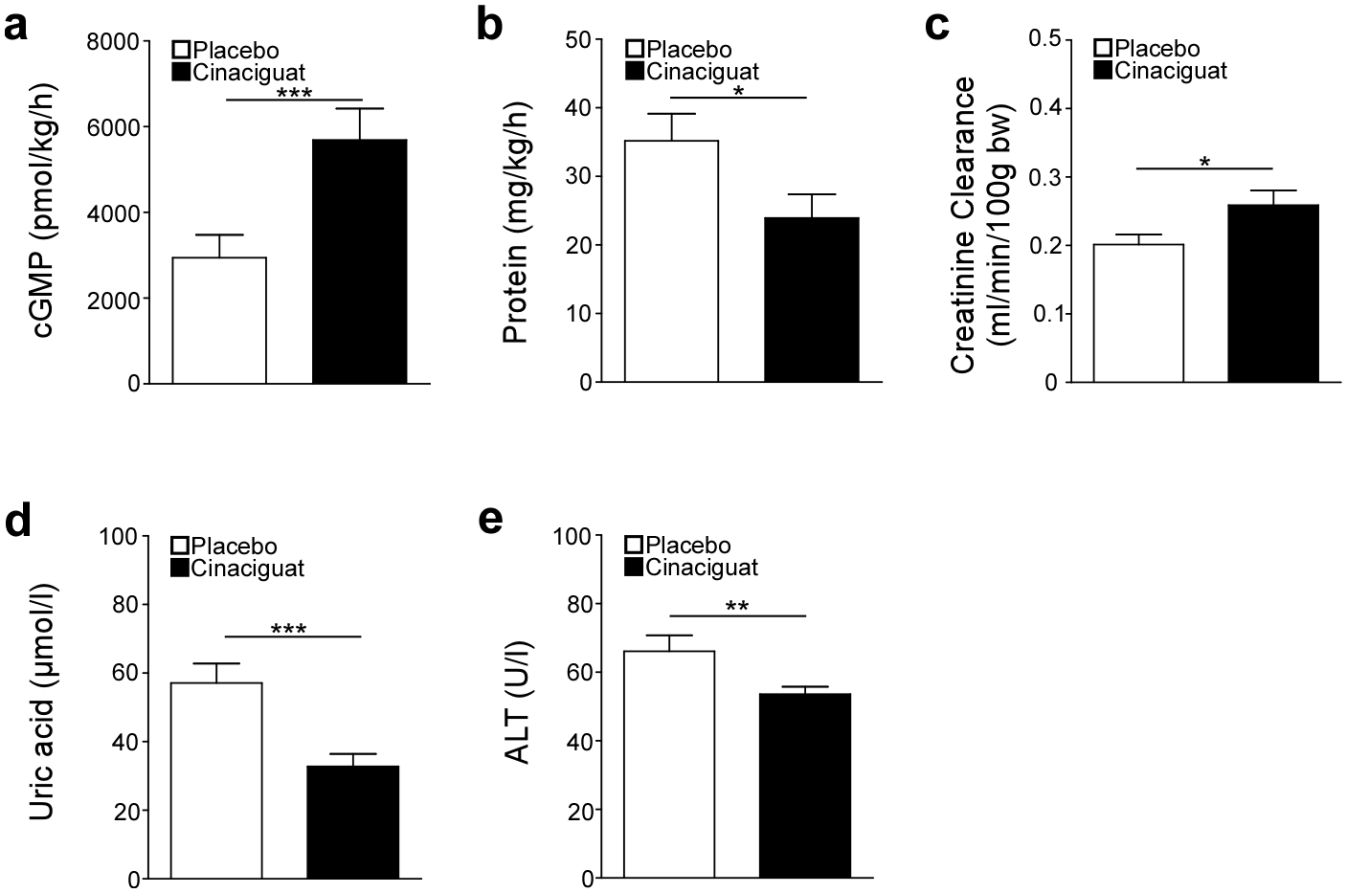
*Effects* of cinaciguat treatment on kidney and liver function. Levels of cGMP (a), protein (b) and creatinine clearance (c) were determined in urine collected over 6 h. Uric acid (d) and activity of alanine aminotransferase (ALT) (e) were determined in plasma collected at the end of the study. *p < 0.05; **p<0.01; ***p < 0.005: Student’s *t*-test (cinaciguat vs. placebo), 15 animals of the placebo group and 16 animals of the cinaciguat group were analyzed.

In week 19, urine was collected over a period of 6 h and renal parameters were analyzed (S1 Table). The cGMP content was significantly higher in the cinaciguat-treated group compared to the placebo group (Fig 2a). Importantly, proteinuria was significantly decreased upon cinaciguat treatment whereas creatinine clearance was significantly increased (Fig 2b and 2c).

At study end blood samples were collected and analyzed (S2 Table). Uric acid and activity of ALT were significantly lower in plasma of cinaciguat-treated animals than in the placebo group (Fig 2d and 2e).

Plasma level of cinaciguat was 216.7 ± 2.2 μg/l in the treatment group at the end of the study.

### Organ weights

The study was terminated 21 weeks after commencement of the treatment and body weights and weights relative to body weight of hearts, left and right ventricle, left and right kidneys were determined (Table 2). Relative kidney weights of cinaciguat-treated animals were significantly lower than in the placebo group whereas there was no difference in relative heart weights between the two groups.

**Table 2:** Relative weights of heart, ventricles and kidneys of animals in placebo group and cinaciguat group at the end of the study. Data are means ± SEM.

Parameter (unit)	Placebo	Cinaciguat
Body weigth (g)	384.1 ± 7.3	367.7 ± 9.2
Heart (g/kg)	4.2 ± 0.1	4.0 ± 0.2
Left ventricle (g/kg)	3.1 ± 0.1	3.0 ± 0.2
Right ventricle (g/kg)	0.6 ± 0.03	0.6 ± 0.03
Left kidney (g/kg)	4.9 ± 0.2	4.3 ± 0.1***
Right kidney (g/kg)	4.9 ± 0.1	4.3 ± 0.2**

**p < 0.01;

***p < 0.005,

Student’s *t*-test (cinaciguat vs. placebo). 10 animals of the placebo group and 15 animals of the cinaciguat group were analyzed.

### Pro-fibrotic and pro-inflammatory biomarkers

**Fig 3:**
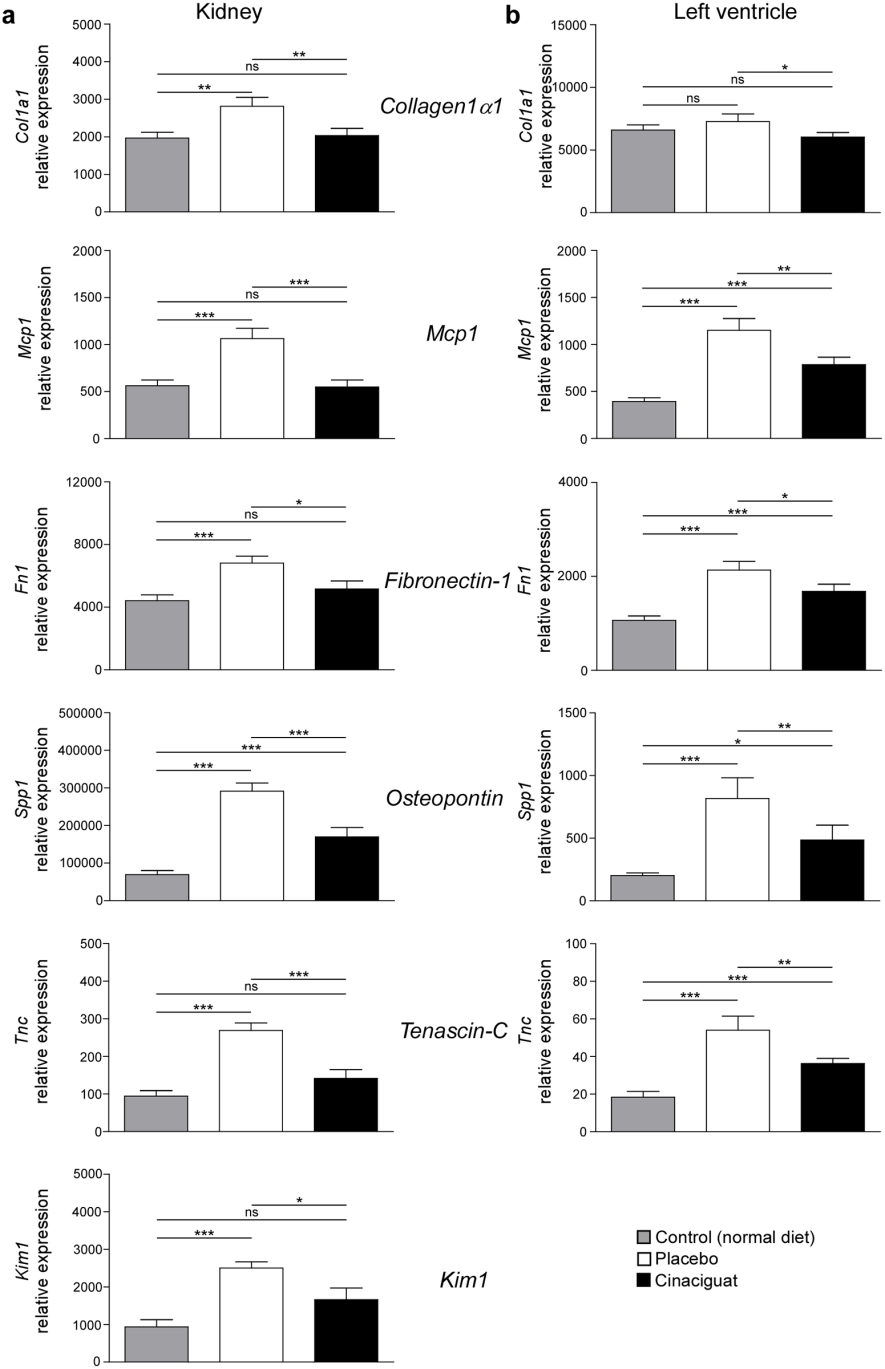
Effect of salt load and cinaciguat treatment on relative mRNA expression of pro-inflammatory and pro-fibrotic biomarker genes. Relative expression of the pro-inflammatory markers *monocyte chemoattractant protein-1* (*Mcp1*) and of the pro-fibrotic markers *collagen1α1* (*Col1a1*), *fibronectin-1* (*Fn1*), *osteopontin* (*Spp1*), and *tenascin-C* (*Tnc*) was assessed in kidney (a) and left ventricle (b) in control animals kept on normal diet and in animals kept on high salt diet (8% NaCl) receiving either placebo or cinaciguat. Data are means ± SEM. *p < 0.05; **p < 0.01; ***p < 0.005: Student’s *t*-test; ns = not significant. 8 animals in the control group, 10 animals of the placebo group and 15 animals of the cinaciguat group were analyzed.

Relative gene expression of the pro-fibrotic biomarkers *collagen1α1* (*Col1a1*), *fibronectin-1* (*Fn1*), *osteopontin* (*Spp1*), *tenascin-C* (*Tnc*) and the pro-inflammatory biomarker *monocyte chemoattractant protein-1* (*Mcp1*) were determined in left ventricle and kidney using quantitative real-time PCRs (Fig 3). *Col1a1*, *Fn1 and Tnc are proteins of the extracellular matrix which is restructured during fibrotic events*. The tissue injury marker *kidney injury molecule-1* (*Kim1*) is upregulated in the cause of renal damage and was used to monitor renal tissue damage.

In the kidneys, relative expression of all tested biomarkers was significantly increased in the placebo group compared to normotensive control animals. Cinaciguat prevented this increase in relative expression of all tested biomarkers resulting in control-like expression levels of all biomarkers, except in the case of *osteopontin*.

In the left ventricle, relative expression of all biomarkers was increased in animals receiving a high salt diet. In the cinaciguat-treated group, expression levels were significantly lower than in the placebo group although they did not reach control niveau. The effect of high salt intake and cinaciguat treatment on the expression of *collagen1α1* was not as pronounced as of the other tested markers.

**Fig 4:**
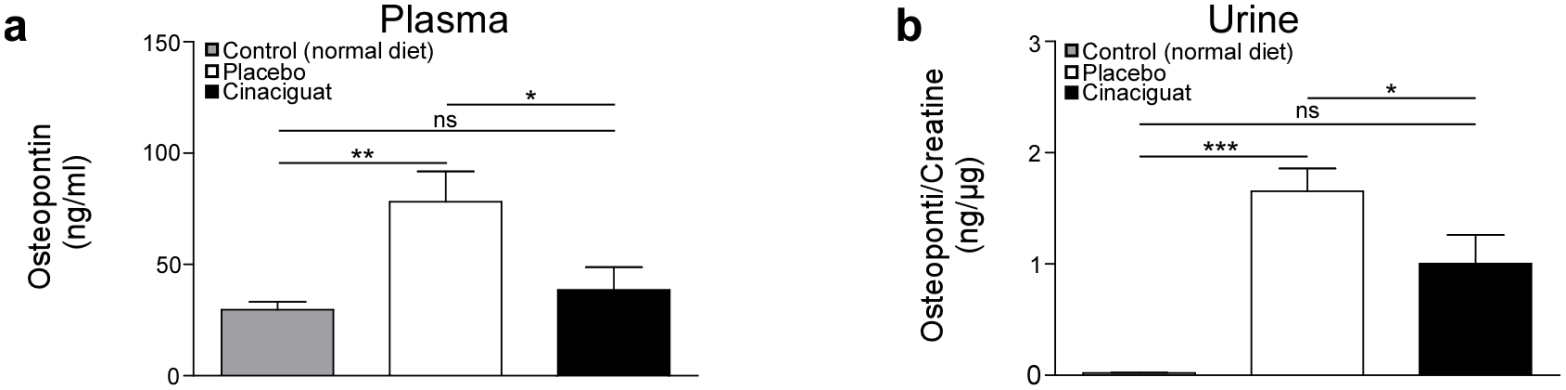
Concentrations of osteopontin in plasma (a) and urine (b) of control animals kept on normal diet and in animals kept on high salt diet (8% NaCl) receiving placebo or cinaciguat, respectively. Data are means ± SEM. *p < 0.05; ***p < 0.005: Student’s *t*-test; ns = not significant. 10 animals in the control group, 10 animals of the placebo group and 15 animals of the cinaciguat group were analyzed.

In addition, osteopontin protein levels were analyzed in urine and plasma samples (Fig 4). Osteopontin was significantly higher in samples from animals of the placebo group than of control animals. Cinaciguat prevented the increase in osteopontin concentrations which were observed in the placebo group. Osteopontin plasma concentration remained at control levels after cinaciguat treatment.

### M-Mode echocardiography

**Fig 5:**
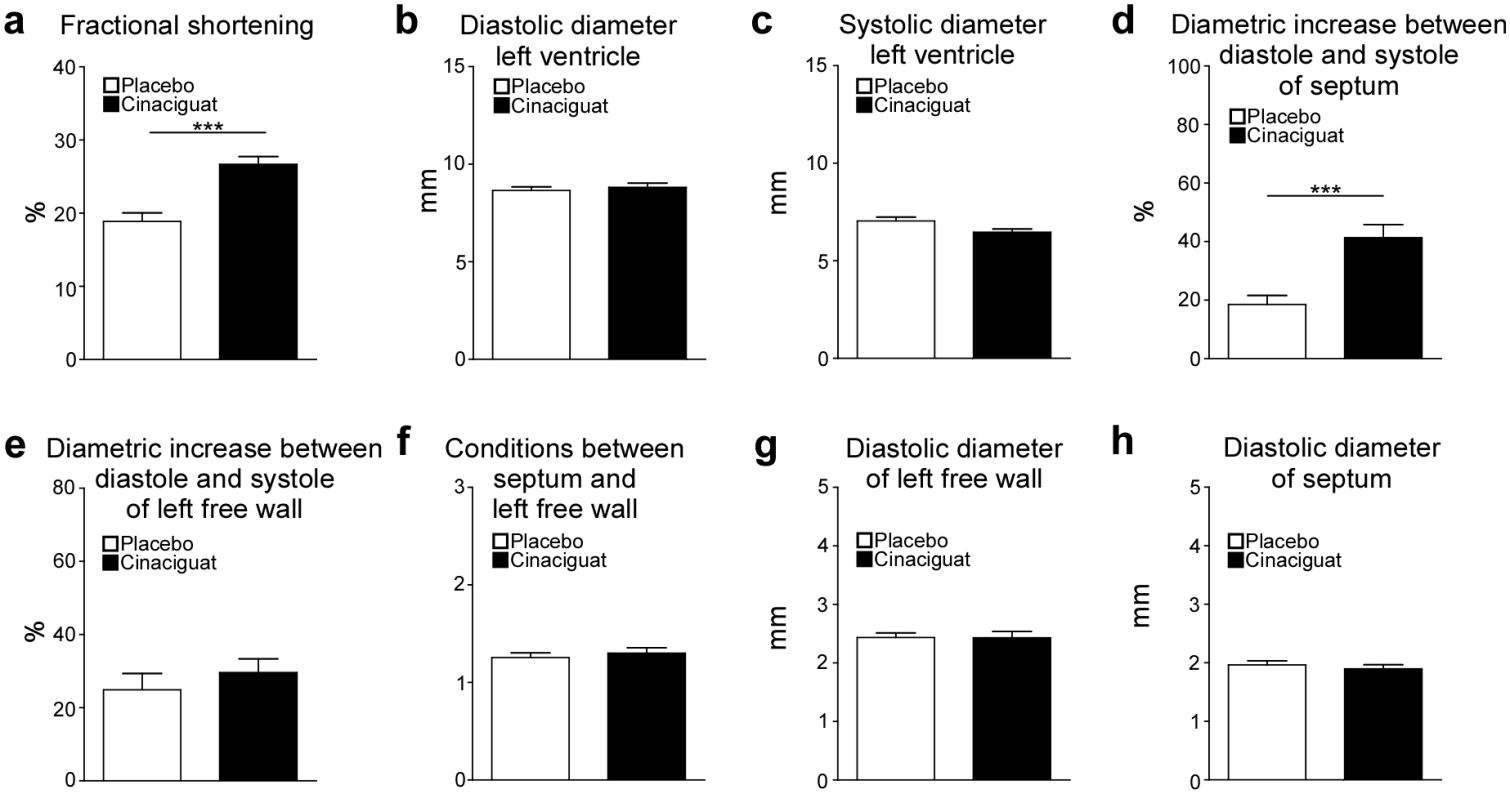
Results of M-Mode echocardiographic analysis performed one week before study end. Fractional shortening (a), diastolic (b) and systolic (c) diameter of the left ventricle, diametric increase between diastole and systole of the septum (d) and left free wall (e), conditions between septum and left free wall (f) as well as diastolic diameter of left free wall (g) and septum (h) were analysed in 14 (placebo) and 16 (cinaciguat) animals. Data are means ± SEM. ***p < 0.005: Student’s t-test (cinaciguat vs. placebo). 14 animals of the placebo group and 16 animals of the cinaciguat group were analyzed.

High resolution echocardiographic studies were performed in week 20 to examine cardiac function. Fractional shortening was significantly increased in the cinaciguat group compared to the placebo group (Fig 5a). Diastolic diameter of left ventricle did not differ between the two groups whereas the systolic diameter was slightly but not significantly shorter in the cinaciguat group then in the placebo group (Fig 5b and 5c). In cinaciguat-treated animals a significant increase in the septum’s diameter was detected between diastole and systole (Fig 5d). However, diametric increase between systole and diastole of the left free wall and conditions between septum and left free wall did not differ between the two groups (Fig 5e and 5f). No differences of diastolic diameter of the left free wall and septum were observed between the two groups (Fig 5g and 5h).

### Histopathology

**Fig 6:**
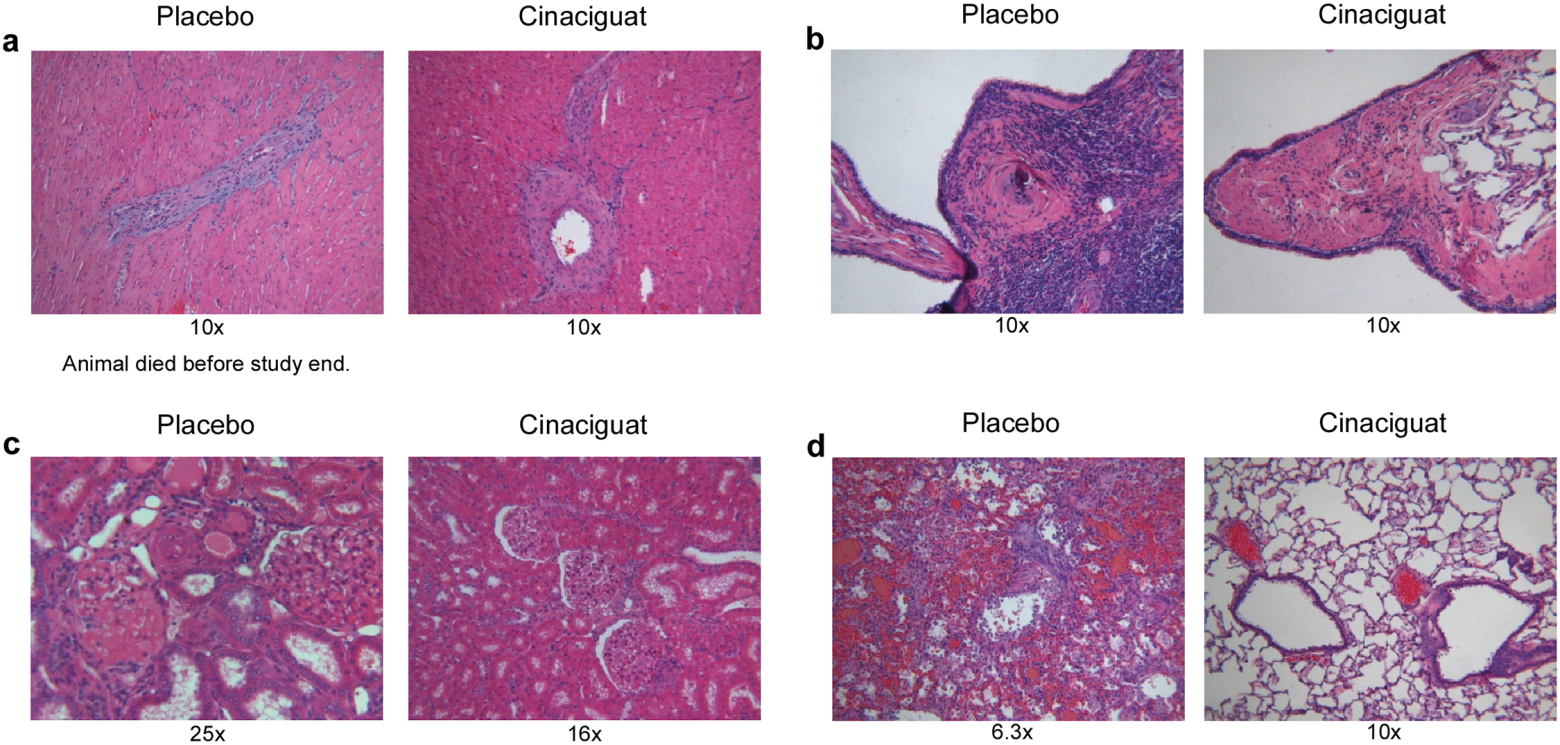
Histological analysis of tissues from rats on a high salt diet with or without (placebo) cinaciguat. Respective sections of myocardial (a) and pulmonary (b) artery, kidney (c) and lung (d) are shown. Sections were HE-stained, kidney sections are additionally PAS-stained. Magnitude is given beyond the respective picture.

Lungs, hearts and kidneys were collected at the end of the study and if possible from animals which died before study end and processed to histopathological examination. Pictures of representative sections are shown in Fig 6. The most affected organ among decedent animals was the kidney with vascular, glomerular and tubular lesions partly up to a severe level (Table 3). Vasculopathy was characterized by hypertrophy of small arteries, perivascular inflammatory cuffing, degeneration or fibrinoid media necrosis, occasional intima proliferation, and extravasation of erythrocytes. Tubulopathy manifested in the occurrence of proteinaceous casts within dilated cortical/medullary tubules, necrosis and exfoliation of tubular epithelial cells, occurrence of basophilic cortical tubules with or without peritubular fibrosis. Glomerulopathy was variously consisting of fibrinoid hyalinization and thrombozation of glomeruli, dilation of glomeruli, activation/thickening of the epithelial parts of Bowman's capsule, protein retention. Together with high scores of myocardial fibrosis and lung inflammation they might demonstrate the pathological reason for interim mortality. Surviving animals at study termination (Table 4) revealed beneficial cinaciguat treatment-related effects in the heart (significantly reduced vasculopathy at p ≤ 0.01, fibrosis, inflammatory infiltration), kidney (significantly reduced vasculopathy at p ≤ 0.01, glomerulopathy, tubulopathy, inflammatory infiltration) and lung tissue (vasculopathy, inflammation).

**Table 3:** Histopathological findings in Dahl/ss rats deceased before study termination. Incidence of non-neoplastic lesions by average grading per organs affected is shown. Necropsy status: terminal sacrifice group, deaths only. Grade 1–5 indicating minimal-slight-moderate-marked-massive, respectively.

Group	Placebo	Cinaciguat
N per group	9	2
**Heart**		
N examined	9	2
Myocardial inflammatory infiltration	2	-
percent of tissues affected	22	-
average grade/tissues affected	1	-
Vasculopathy	6	1
percent of tissues affected	67	50
average grade/tissues affected	1.5	1.0
Myocardial fibrosis	8	2
percent of tissues affected	89	100
average grade/tissues affected	1.9	2.0
**Lung**		
N examined	9	2
Peri-/Vasculitis	6	1
percent of tissues affected	67	50
average grade/tissues affected	1.2	2.0
Vasculopathy	4	1
percent of tissues affected	44	50
average grade/tissues affected	1.5	1.0
**Kidneys**		
N examined	9	2
Glomerulopathy	9	2
percent of tissues affected	100	100
average grade/tissues affected	3.9	4.0
Tubulopathy	9	2
percent of tissues affected	100	100
average grade/tissues affected	4.0	4.0
Vasculopathy	9	1
percent of tissues affected	100	50
average grade/tissues affected	3.2	1.0
Inflammatory Infiltration	8	-
percent of tissues affected	89	0
average grade/tissues affected	2.0	-

**Table 4:** Histopathological findings in Dahl/ss at terminal sacrifice. Incidence of non-neoplastic lesions by average grading per organs affected is shown. Necropsy status: terminal sacrifice group, except deaths. Grade 1–5 indicating minimal-slight-moderate-marked-massive, respectively.

Group	Placebo	Cinaciguat
N per group	10	17
**Heart**		
N examined	10	17
Myocardial inflammatory infiltration	4	3
percent of tissues affected	40	18
average grade/tissues affected	1.3	1.0
Vasculopathy	8	2**
percent of tissues affected	80	12
average grade/tissues affected	1.8	1.5
Myocardial fibrosis	6	6
percent of tissues affected	60	35
average grade/tissues affected	1.3	1.0
**Lung**		
N examined	10	17
Alveolar macrophages/foam cells	6	5
percent of tissues affected	60	29
average grade/tissues affected	1.0	1.4
Peri-/Vasculitis	4	4
percent of tissues affected	40	24
average grade/tissues affected	1.3	1.5
Vasculopathy	5	5
percent of tissues affected	50	29
average grade/tissues affected	1.2	1.0
**Kidneys**		
N examined	10	17
Glomerulopathy	10	12
percent of tissues affected	100	71
average grade/tissues affected	3.1	2.1
Tubolupathy	10	17
percent of tissues affected	100	100
average grade/tissues affected	3.2	2.7
Vasculopathy	10	5**
percent of tissues affected	100	29
average grade/tissues affected	3.3	3.4
Inflammatory infiltration	10	15
percent of tissues affected	100	88
average grade/tissues affected	2.5	1.2

**p ≤ 0.01,

One-sided Fisher Exact Test.

## Discussion

Dysfunction of the protective NO/sGC/cGMP-signalling pathway is involved in the development of severe cardiovascular disease like hypertension-induced chronic renal failure [6, 8, 37, 38]. Oxidative stress is increased in particular in chronic renal failure and is characterized by increased formation of ROS [9, 39]. These reactive molecules interfere with sGC signalling by scavenching of NO and oxidizing the prosthetic heme group to the NO-insensitive Fe^3+^ state [26, 40, 41]. Oxidation results in a reduced affinity of the heme group to the binding pocket and is followed by a loss of the heme group [13, 17, 20, 28, 42]. The NO- and heme-independent sGC activators such as cinaciguat selectively activate the heme free form of sGC and thereby target the disease associated enzyme.

This study investigated the therapeutic effect of chronic activation of heme free sGC by cinaciguat in a long-term study using Dahl/ss rats. This rat strain develops severe hypertension and chronic renal failure upon high salt intake [30]. Oxidative stress is thought to be involved in the development of hypertension and accompanied by renal organ damage [43–45]. Furthermore, sensitivity of sGC towards NO is reduced in kidneys of salt-loaded Dahl/ss rats which presumably is due to an increased pool of heme free sGC in hypertensive animals compared to respective healthy controls [46].

Oral treatment with cinaciguat significantly reduced the salt-induced increase of blood pressure and heart rate as observed in the placebo group. A similar blood pressure lowering effect was also observed in other experimental animal models upon oral administration of cinaciguat [18, 23, 25, 34]. The therapeutic effect of cinaciguat was particularly evident in the dramatically improved survival in the treated group compared to placebo. Furthermore, the results of the echocardiographic studies point to an improved cardiac function. Fractional shortening and diameter increase of the septum between systole and diastole was elevated in the cinaciguat group compared to the placebo group indicating an improved contractility of the heart upon treatment with cinaciguat.

The most striking effect of cinaciguat was shown on kidney function and morphology. From a clinical point of view, preservation of GFR and reduction of urinary protein excretion are the most important renal biomarkers, because improvement of GFR and reduction of urinary protein excretion are associated in clinical phase III trials with improved all-cause mortality, reduction of need for renal replacement therapy and reduction of doubling of serum creatinine—the typical composite phase III endpoint usually requested by legal authorities like the FDA for drug approval. Exactly these key parameters and mortality were improved by long-term treatment with cinaciguat in Dahl/ss rats.

This fits well with the observed reduction in uric acid, which is considered as an independent cardiovascular and renal risk predictor [47] and hence these data are of high clinical relevance from a translational point of view.

Comparable effects regarding improved survival, lowering of blood pressure and heart rate, reduced ANP levels, improved fractional shortening and improved renal function were observed for the sGC stimulator riociguat, a compound that also targets sGC but solely stimulates the heme containing reduced redox form of the enzyme in synergism with NO [48].

Plasma concentration of ALT was reduced by cinaciguat indicating a cinaciguat-mediated improvement of liver function. This result is in line with studies showing that the therapeutic effect observed for the structurally related sGC activator BAY 60–2770 was beneficial in a different experimental model of liver fibrosis [49].

Anti-fibrotic and anti-inflammatory effects of long-term treatment with cinaciguat were investigated with the use of biomarkers. In this set of experiments organs from control animals receiving a diet with normal salt content were analyzed in addition to the two study groups. Biomarker analyses revealed that cinaciguat prevented or decreased fibrotic and inflammatory events in the hearts and kidneys of animals on a high salt diet. The expression levels of all investigated markers were higher in the placebo group than in animals receiving normal salt diet. Noteworthy, expression of the tested biomarkers was lower in the cinaciguat group than in the placebo group. The extracellular matrix is a dynamic superstructure consisting of macromolecules like fibronectin or collagens but its composition is altered in the cause of fibrotic events [50]. The extracellular matrix glycoprotein tenascin-C is upregulated during remodeling and inflammatory processes [51]. Osteopontin’s expression is accelerated in the case of myocardial fibrosis and by mechanical stress due to pressure overload [52, 53]. Furthermore, a correlation between increased plasma concentrations in patients with heart failure and disease stage and mortality was observed [53]. Kim1 is not detectable under physiological conditions but is upregulated due to ischemic and toxic damage in the kidney [54]. Mcp1 is involved in inflammatory events and in the formation of atherosclerotic plaques [55]. The profound anti-inflammatory and anti-fibrotic effect of cinaciguat treatment was demonstrated by a similar relative expression of *collagen 1α1*, *Mcp1*, *Fn1*, *Tnc* and *Kim1* in the kidneys of control and cinaciguat-treated animals. In addition, the histopathological examination of organs from descedent animals revealed that fibrotic and inflammatory events especially in the heart and kidney were the cause of death. These pathological lesions were markedly decreased by cinaciguat. Wang et al. showed in an experimental model for chronic glomerulosclerosis with associated fibrosis that treatment with the NO-independent and heme-dependent sGC stimulator BAY 41-2272 resulted in a reduction of the expression of pro-fibrotic markers like fibronectin [56]. Furthermore, immunohistochemistry in the same experimental model revealed that BAY 41-2272 prevents the accumulation of extracellular matrix. These results and the results presented in this paper indicate that NO-independent sGC activation could result in protective effects on fibrotic events.

At the end of this study similar plasma levels of cinaciguat were measured as in a study with the 5/6 nephrectomy model in which cinaciguat prevented an increase in blood pressure and hypertrophy of the left heart and showed a protective effect on kidney function and morphology [25].

A limitation of the study is that the food intake was not monitored on the individual animal level. Lower weight gain in the cinaciguat group at the beginning of the study indicates decreased food intake which was highly likely due to the different taste of the compound in the diet. The lower amount of food might have decreased the amount of salt ingested by the animals and might have increased dose variability of the drug resulting in variability of plasma drug concentrations. However, at the end of the study there was no difference in body weight between the placebo and the treatment group and the measurement of drug levels at study end confirmed that our approach resulted in drug levels around target concentration [25]. Nevertheless, it is highly recommended to measure food intake per animal in similar future studies or to perform pair feeding experiments to ensure comparable food and salt intake at all time points of the study. Furthermore, our protocol to measure cardiac function by echocardiography did not include measurements of stroke volume and cardiac output. These measurements would be valuable additions to the performed assessments and should be included in future studies.

Taken together, the results of this study clearly demonstrate that long-term activation of heme free sGC with cinaciguat leads to renal protection. This effect was demonstrated by a noticeable reduction of mortality, improved renal function—in particular protein excretion and preservation of GFR -, an ameliorated increase in blood pressure and reduced inflammatory and fibrotic biomarkers in heart and kidney. In line with previous experimental and clinical studies, these results show the promising potential of cinaciguat to treat renal diseases by targeting the diseased associated heme free form of sGC.

## Supporting Information

S1 ARRIVE Checklist ARRIVE Checklist: Scan of the ARRIVE Checklist in which page numbers of the originally submitted manuscript are given. (PDF)

S1 Table Effects of long-term treatment (21 weeks) with cinaciguat on renal parameters. Data are means ± SEM. *p < 0.05 (cinaciguat vs. placebo).
(DOCX)

S2 Table Effects of long-term treatment (21 weeks) with cinaciguat on plasma parameters. Blood samples were taken at study end and parameters were measured. Data are means ± SEM. CK: creatine kinase; LDH: lactate dehydrogenase; GLDH: glutamate dehydrogenase; AST: aspartate amino transferase; AP: alkaline phosphatase; ANP: atrial natriuretic peptide.
(DOC)
