# Supplementary material for: Chronic Activation of Heme Free Guanylate Cyclase Leads to Renal Protection in Dahl Salt-Sensitive Rats
Source: PLoS One. 2015 Dec 30;10(12):e0145048. doi: 10.1371/journal.pone.0145048 (PMC4700984; doi:10.1371/journal.pone.0145048)
Supplement: S1 Table — Data are means ± SEM. *p < 0.05 (cinaciguat vs. placebo). (DOCX) [file pone.0145048.s002.docx]

S1 Table

| Parameter (unit) | Placebo | Cinaciguat |
| --- | --- | --- |
| Diuresis (ml/kg/h) | 5.7 ± 0.6 | 4.2 ± 0.6* |
| Creatinin excretion (µmol/kg/h) | 7.5 ± 0.4 | 8.2 ± 0.5 |
| Urea excretion (µmol/kg/h) | 469.3 ± 62.3 | 450.9 ± 78.0 |
| Sodium excretion (µmol/kg/h) | 968.9 ± 170.6 | 865.7 ± 122.9 |
| Potassium excretion (µmol/kg/h) | 201.0 ± 25.6 | 172.2 ± 20.4 |
