# Supplementary material for: Chronic Activation of Heme Free Guanylate Cyclase Leads to Renal Protection in Dahl Salt-Sensitive Rats
Source: PLoS One. 2015 Dec 30;10(12):e0145048. doi: 10.1371/journal.pone.0145048 (PMC4700984; doi:10.1371/journal.pone.0145048)
Supplement: S2 Table — Blood samples were taken at study end and parameters were measured. Data are means ± SEM. CK: creatine kinase; LDH: lactate dehydrogenase; GLDH: glutamate dehydrogenase; AST: aspartate amino transferase; AP: alkaline phosphatase; ANP: atrial natriuretic peptide. (DOC) [file pone.0145048.s003.doc]

S2 Table

| Parameter (unit) | Placebo | Cinaciguat |
| --- | --- | --- |
| Creatinin (µmol/l) | 63.6 ± 5.5 | 54.8 ± 2.1 |
| Urea (mmol/l) | 8.5 ± 1.6 | 8.8 ± 1.0 |
| CK (U/l) | 319.1 ± 35.8 | 293.2 ± 26.4 |
| LDH (U/l) | 597.5 ± 86.7 | 471.4 ± 65.8 |
| GLDH (U/l) | 17.3 ± 3.6 | 21.8 ± 2.6 |
| AST (U/l) | 86.9 ± 8.3 | 82.9 ± 6.2 |
| AP (U/l) | 72.7 ± 5.3 | 75.1 ± 3.1 |
| Protein (g/l) | 67.8 ± 1.3 | 68.8 ± 1.4 |
| ALB (g/l) | 28.6 ± 0.5 | 30.5 ± 0.6* |
| ANP (pg/ml) | 287.2 ± 51.0 | 231.6 ± 36.9 |
| cGMP (pmol/ml) | 33.4 ± 7.5 | 42.9 ± 6.2 |
